# Salmonellosis outbreak with novel *Salmonella enterica* subspecies *enterica* serotype (11:z41:e,n,z15) attributable to sesame products in five European countries, 2016 to 2017

**DOI:** 10.2807/1560-7917.ES.2019.24.36.1800543

**Published:** 2019-09-05

**Authors:** Anika Meinen, Sandra Simon, Sangeeta Banerji, Istvan Szabo, Burkhard Malorny, Maria Borowiak, Sead Hadziabdic, Natalie Becker, Petra Luber, Dorothee Lohr, Carolin Harms, Anita Plenge-Bönig, Kassiani Mellou, Georgia Mandilara, Joël Mossong, Catherine Ragimbeau, Pierre Weicherding, Patrick Hau, Daniela Dědičová, Lucie Šafaříková, Satheesh Nair, Timothy J Dallman, Lesley Larkin, Jacquelyn McCormick, Elizabeth De Pinna, Ettore Severi, Saara Kotila, Taina Niskanen, Valentina Rizzi, Domenico Deserio, Antje Flieger, Klaus Stark

**Affiliations:** 1 Robert Koch Institute, Department for Infectious Disease Epidemiology, Berlin, Germany; 2 These authors contributed equally; 3 Robert Koch Institute, Department of Infectious Diseases, National Reference Centre for Salmonella and Other Bacterial Enteric Pathogens, Wernigerode, Germany; 4 German Federal Institute for Risk Assessment (BfR), Department for Biological Safety, Berlin, Germany; 5 Federal Office of Consumer Protection and Food Safety, Crisis Unit Office, Foodborne Outbreaks, Prevention, Berlin, Germany; 6 Baden-Wuerttemberg State Health Office, Stuttgart, Germany; 7 Institute for Hygiene and Environment Hamburg, Department of Microbiology, Hamburg, Germany; 8 Institute for Hygiene and Environment Hamburg, Infectious Disease Surveillance Unit, Hamburg, Germany; 9 Hellenic National Public Health Organization, Athens, Greece; 10 National School of Public Health, National Reference Centre for Salmonella, Athens, Greece; 11 Laboratoire National de Santé, Département de Microbiologie, Dudelange, Luxembourg; 12 Direction de la Santé, Luxembourg, Luxembourg; 13 Státní zdravotní ústav (National Institute of Public Health), Prague, Czech Republic; 14 Public Health England, London, United Kingdom; 15 European Centre for Disease Prevention and Control (ECDC), Surveillance and Response Support, Stockholm, Sweden; 16 European Food Safety Authority, Parma, Italy

**Keywords:** food-borne infections, outbreaks, Salmonella, Salmonella Vari, salmonellosis, sesame

## Abstract

In spring 2016, Greece reported an outbreak caused by a previously undescribed *Salmonella* *enterica* subsp. *enterica* serotype (antigenic formula 11:z41:e,n,z15) via the Epidemic Intelligence Information System for Food- and Waterborne Diseases and Zoonoses (EPIS-FWD), with epidemiological evidence for sesame products as presumptive vehicle. Subsequently, Germany, Czech Republic, Luxembourg and the United Kingdom (UK) reported infections with this novel serotype via EPIS-FWD. Concerned countries in collaboration with the European Centre for Disease Prevention and Control (ECDC) and European Food Safety Authority (EFSA) adopted a common outbreak case definition. An outbreak case was defined as a laboratory-confirmed notification of the novel *Salmonella* serotype. Between March 2016 and April 2017, 47 outbreak cases were notified (Greece: n = 22; Germany: n = 13; Czech Republic: n = 5; Luxembourg: n = 4; UK: n = 3). Whole genome sequencing revealed the very close genetic relatedness of isolates from all affected countries. Interviews focusing on sesame product consumption, suspicious food item testing and trace-back analysis following *Salmonella* spp. detection in food products identified a company in Greece where sesame seeds from different countries were processed. Through European collaboration, it was possible to identify and recall sesame spread as one contaminated food item serving as vehicle of infection and trace it back to its origin.

## Background

*Salmonella* spp. is responsible for the majority of food-borne outbreaks in Europe [1]. Between 15 March and 30 May 2016, the Greek National Reference Laboratory for *Salmonella* and *Shigella* (NRLSS) in Vari, a suburb of Athens, detected 16 *Salmonella enterica* subsp. *enterica* isolates with the antigenic formula 11:z41:e,n,z15 sharing an indistinguishable PFGE profile. This combination of antigens is not listed in the current ninth edition of the White-Kauffman–Le Minor scheme [2,3]. The World Health Organization (WHO) Collaborating Centre for Reference and Research on *Salmonella* at Institut Pasteur in Paris, France, confirmed the antigenic profile 11:z41:e,n,z15 as a novel *Salmonella* serotype [4]. The proposed name of the novel serotype is *Salmonella* Vari.

Initial epidemiological investigations did not reveal any apparent link between the cases [4]. First cases had disease onset in March 2016, around the beginning of the Lent season of Orthodox Easter when various sesame products are traditionally served. Results of a case–case study provided evidence for tahini, a paste made from hulled, ground and toasted sesame seeds, being the most probable vehicle of infection in Greece. However, it was not possible to identify a single product trademark or a single place of purchase of the tahini. No food isolate had been recovered for testing.

## Multi-country outbreak detection

An urgent inquiry (UI-358) was launched by Greece in the European Centre for Disease Prevention and Control (ECDC)’s Epidemic Intelligence Information System for Food- and Waterborne Diseases and Zoonoses (EPIS-FWD) [5] on 10 May 2016 to ask if other countries also detected this novel serotype. Between May 2016 and April 2017, Germany, Czech Republic, Luxembourg and the United Kingdom (UK) also reported salmonellosis cases infected with the novel serotype through EPIS-FWD. ECDC called for a multi-country investigation and formed an outbreak investigation team comprised of experts from public health institutes at national and European levels. National- and European-level food safety agencies were also involved in the outbreak investigation.

Here we describe how the outbreak investigation was conducted and what it unveiled. We also address what control measures were put in place in response to the outbreak.

## Methods

### Epidemiological investigation and sampling of food samples

#### Case definition

The multi-country outbreak investigation team decided on a common outbreak case definition. Outbreak cases were defined as laboratory-confirmed notifications of *Salmonella enterica* subspecies *enterica* with the antigenic formula 11:z41:e,n,z15 (or corresponding in silico serotype/multilocus sequence type retrieved from whole genome sequencing (WGS) data) between 1 March 2016 and 30 April 2017.

Cases were interviewed using national questionnaires. The questionnaires included questions on travel to Greece or visits from relatives or friends from Greece, and sesame consumption in the week before disease onset.

#### Food samples

Sesame products mentioned in more than one interview were, when available from cases’ households, sampled by the responsible food and veterinary authorities and analysed for *Salmonella*. Additionally, official control samples from the retail shops where the cases had bought these products were taken and analysed in official control laboratories. As part of the outbreak investigation, staff from the German Federal Institute for Risk Assessment (BfR) and the Robert Koch Institute (RKI), both in Berlin, Germany, purchased food items of the same type from a German online shop or in retail shops in Berlin without direct connection to the cases. These unofficial samples were directly sent to the National Reference Laboratory (NRL) at BfR and analysed for *Salmonella*.

Detection of the novel *Salmonella* serotype in food taken as part of companies’ own routine checks (samples taken by the food business operator to ensure compliance with food safety requirements) or official routine control samples was communicated among the outbreak team.

### Microbiology and whole genome sequencing analysis

All *Salmonella enterica* subspecies *enterica* serotype 11:z41:e,n,z15 human isolates from Greece, Germany, Czech Republic, Luxembourg and the UK underwent phenotypic serotyping by slide agglutination [2,3]. The serotype of the UK isolates was also confirmed by in silico serotyping based on WGS data with SeqSero version 1 [6]. Since this particular antigenic formula had not been listed in the White-Kauffman-Le Minor scheme, one respective strain from Greece was sent to the WHO Collaborating Centre for Reference and Research on *Salmonella* at Institut Pasteur for confirmation.

The food items sampled in Germany were tested for *Salmonella* spp. according to ISO 6579:2002/Amd 1:2007 [7]. For the enumeration of *Salmonella* in the food samples, the direct plating method was performed by spread plating 0.1 ml and 1 ml of each sample from the 1:10 dilution in triplicates onto XLD agar plates, respectively.

ECDC initiated WGS for isolates from Greece, Germany, Czech Republic and Luxembourg to assess their relationship. Public Health England (PHE) performed WGS of the UK strains. Paired-end, short-read sequencing via MiSeq or HiSeq (Illumina, San Diego, California, United States) was performed either by a commercial provider that was coordinated and funded by ECDC, or by the public health institutes of the countries themselves.

The National Reference Centre for Salmonella and Other Bacterial Enteric Pathogens at RKI conducted the multi-country sequence analysis. Raw reads (Germany) or assembled genomes (Luxembourg) were provided by the individual partners or by ECDC (Greece and Czech Republic). Raw reads from the UK strains were downloaded from the Sequence Read Archive (SRA), respectively. The seven-locus multilocus sequence typing (MLST) based on whole genome data according to the scheme of Achtman et al. [8], as well as gene-by-gene comparison, were conducted using Ridom SeqSphere^+^ version 5.1.0_(2018-06) [9]. Sequence data were processed using the programme’s default settings for quality control and allele calling. Raw reads were de novo assembled by the implemented assembly algorithm (Velvet version 1.1.04). The applied ad hoc core genome (cg)MLST scheme for *Salmonella enterica* established at RKI comprises 2,143 core genome loci [10]. Genomes of 24 human isolates from Greece, Germany, Czech Republic, Luxembourg and the UK, as well as sequences from four food isolates from Germany, Luxembourg and the UK were analysed with this scheme. For cgMLST analysis, a minimum of 95% good targets was required. Sequence reads from German and UK isolates, both from humans and food, are available under the European Nucleotide Archive (ENA)/National Center for Biotechnology Information (NCBI) accession numbers PRJEB27505 (ERS2589577–ERS2589590) and PRJNA248792 (SRR5413117–SRR5413122), respectively. Sequence data from the Luxembourgish human strains and from one exemplary human strain from Greece are available on Enterobase [11].

### Trace-back investigations

When *Salmonella* spp. was detected in food, trace-back investigations were initiated. Information about the implicated food sources reported via the Rapid Alert System for Food and Feed (RASFF) was used by the European Food Safety Authority (EFSA) to perform the food traceability investigation at the European Union (EU) level. Companies identified by trace-back analysis were visited by the respective competent authorities who assessed production procedures and hygiene.

## Results

### Epidemiological investigation

Applying the joint outbreak case definition, 47 outbreak cases were notified between 1 March 2016 and 30 April 2017 (Greece: n = 22; Germany: n = 13; Czech Republic: n = 5; Luxembourg: n = 4; UK n = 3) (Table 1). Five of 34 cases with clinical information available were asymptomatic. Of 26 cases with information available on hospitalisation, 12 were hospitalised [12].

**Table 1:** Description of salmonellosis outbreak cases with novel *Salmonella enterica* subspecies *enterica* serotype (11:z41:e,n,z15), five European countries, 1 March 2016–30 April 2017 (n = 47)^a^

Country	Number of cases during the outbreak period	Number of female cases	Median age (years)	Age range (years)	Number of asymptomatic cases/cases with information on symptoms available
Greece	22	8	3	0–62	1/19
Germany	13	9	30	2–75	1/11
Czech Republic	5	3	3	0–41	NA
Luxembourg	4	3	51	31–59	3/4
United Kingdom	3	3	61	6–70	NA
**Total**	**47**	**26**	**8**	**0–75**	**5/34**

NA: no information available.

^a^ In the aftermath of the outbreak investigation, seven additional infections with the novel serotype were notified between 1 May and 30 September 2017 (France: n = 2; Greece: n = 2; Germany: n = 1; Luxembourg: n = 1; Serbia: n = 1).

Based on the findings of the investigations in Greece, interviews in Germany and Luxembourg focused on the consumption of sesame products. In Germany, nine of 13 cases were exposed within the country. Of the nine, five agreed to detailed interviews conducted by RKI between February and May 2017. In Luxembourg, all four cases were interviewed. In total, seven of nine interviewed patients (5/5 from Germany; 2/4 from Luxembourg) reported consuming a particular brand of sweet sesame spread.

In the aftermath of the outbreak investigation, seven additional infections with the novel serotype were notified between 1 May and 30 September 2017 (France: n = 2; Greece: n = 2; Germany: n = 1; Luxembourg: n = 1; Serbia: n = 1).

#### Food testing

In March 2016, sushi containing sesame seeds tested positive for *Salmonella* *enterica* subspecies *enterica* serotype 11:z41:e,n,z15 (which was at that time, designated as ‘*Salmonella* unnamed’) via a company’s own check at a private testing laboratory in the UK, but follow-up testing did not identify *Salmonella* in the constituent ingredients (Table 2). This finding was communicated in response to the Early Warning Response System (EWRS) notification on the publication of the ECDC Rapid Risk Assessment in March 2017 [12].

**Table 2:** Overview of food specimens that tested positive for the novel *Salmonella enterica* subsp. *enterica* serotype (11:z41:e,n,z15), Germany, Luxembourg and the United Kingdom, 21 March 2016–4 May 2017

Number	Country	Product	Sampling location	Sampling date	Packaging	Origin of seeds	RASFF	Date serotype information available to multi-country outbreak investigation team
**1**	Germany	Sesame seeds^a^	Company’s own routine check	19 October 2016	540 bags of sesame seeds (each bag = 22.68 kg)	Nigeria	RASFF 2017.0221	14 December 2016
**2**	Germany	Sesame spread^b^	Household of case	21 March 2017	Sealed jar	Sudan	RASFF 2017.0408	5 April 2017
**3**	Germany	Sesame spread^b^	Retail shop^c^	21 March 2017	Sealed jar	Sudan	RASFF 2017.0408	5 April 2017
**4**	Germany	Sesame spread^a,b^	Online shop	24 March 2017	Sealed jar	Sudan	Not mentioned	29 March 2017
**5**	Germany	Sesame spread^b^	Online shop	24 March 2017	Sealed jar	Sudan	Not mentioned	29 March 2017
**6**	Germany	Sesame spread^b^	Online shop	24 March 2017	Sealed jar	Sudan	Not mentioned	29 March 2017
**7**	Germany	Sesame spread^b^	Retail shop (Berlin)	22 March 2017	Sealed jar	Sudan	Not mentioned	NA
**8**	Germany	Sesame spread^b^	Retail shop (Berlin)	23 March 2017	Sealed jar	Sudan	Not mentioned	NA
**9**	Germany	Sesame spread^b^	Household of case	4 May 2017	Opened jar	Sudan	Not mentioned	17 May 2017
**10**	Luxembourg	Sesame spread^a,b^	Retail shop	3 May 2017	Sealed jar	Sudan	Not mentioned	24 April 2017
**11**	United Kingdom	Sushi containing sesame^a^	Company’s own check	Isolate received at Reference Laboratory 21 March 2016	Unknown	Unknown	None – further testing did not confirm *Salmonella* contamination of product	3 April 2017

NA: no information available; RASFF: Rapid Alert System for Food and Feed.

^a^ Included in whole genome sequencing (WGS) analysis.

^b^ All from the same lot.

^c^ Reference sample for Number 2. Sample was purchased from the same retail shop that Number 2 purchased the product from.

In December 2016, one delivery of sesame seeds from Greece to Germany tested positive for *Salmonella enterica* serotype 11:z41:e,n,z15 during a company’s own routine check in Germany. The company routinely examines every new batch of sesame seeds delivered to their facilities before using them in food production. The whole contaminated batch was sent back to the distributing company in Greece. The findings were communicated via RASFF 2017.0221 [13]. The *Salmonella* isolate was sent for serotyping to the Institute for Hygiene and Environment in Hamburg and from there, the results were forwarded to RKI.

Official samples were taken in households of two German cases: one was taken in March 2017 from the household of a school aged child and one in May 2017 from a person in their 60s. One of these two jars was still sealed. A reference sample was taken from the retail shop where the jar was purchased from.

Another three jars were ordered online by BfR and an additional two jars were bought at retail shops in Berlin by RKI.

In total, eight jars of the sesame spread were collected in Germany. In Luxembourg, one jar was bought from a retail shop. All nine jars were from the same lot, the only one on the market at that time, and *Salmonella enterica* subsp. *enterica* serotype (antigenic formula 11:z41:e,n,z15) could be recovered from each of them. Of the eight sealed jars, three were used to quantify *Salmonella* spp.. The number of *Salmonella* spp. determined by direct plating ranged from 77 to 160 colony forming units (cfu)/g sesame spread in three unopened jars. Results were communicated via RASFF 2017.0408 [14].

### Microbiology and whole genome sequencing analysis

**Figure 1:**
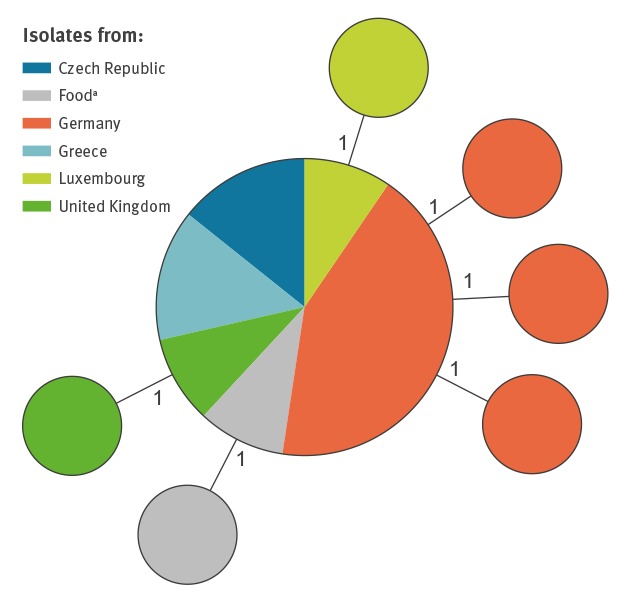
Minimum spanning tree showing genetic relatedness within the novel *Salmonella enterica* subsp. *enterica* serotype (11:z41:e,n,z15), five European countries, 1 March 2016–30 April 2017

^a^ Sesame spread, sesame seeds and sushi containing sesame.

Whole genome sequences from 24 human isolates (Germany: n = 12; Czech Republic: n =3; Greece: n = 3; Luxembourg: n = 3; UK: n =3) and four food isolates (sesame spread: n =2; sesame seeds: n =1; sushi: n =1) were analysed. The average per-base coverage (assembled) ranged from 27- to 116-fold (median 60.5-fold) for the 28 WGS datasets. All samples belonged to seven-locus sequence type ST2914. For gene-by-gene comparison by cgMLST applying the RKI ad hoc scheme based on 2,143 cg-loci, a minimum of 95% good targets was required. The median percentage of good targets was 99.8 (Supplementary Table S1). CgMLST revealed one distinct cluster with a maximum pairwise distance of 2 cgMLST alleles between any two isolates (Figure 1).

### Trace-back and trace-forward investigations

The sesame spread positive for *Salmonella enterica* subsp. *enterica* (antigenic formula 11:z41:e,n,z15) had a best before date of 1 February 2018 and was produced in Greece by company A with sesame seeds originating from Sudan. These seeds were harvested in Sudan in June 2015, arriving at company A in Greece in November 2015. Tahini from these seeds was produced on 18 March 2016. It was stored in plastic pallet tanks until 21 March 2016 when part of it was mixed with the other sesame spread ingredients: sugar, cottonseed oil and soya lecithin. The resulting spread was pumped through a closed pipeline system into a stainless steel holding tank at ca 45 °C. The product was filled into glass jars on 3 consecutive days, 21 to 23 March 2016. The jars had undergone ultraviolet (UV) treatment before filling (without the caps, RASFF 2017.0408-fup15 [14]). The remaining sesame seeds from the lot from Sudan were used for the production of sesame oil, and hulled as well as natural roasted seeds.

Trace-forward investigations showed that the sesame spread had been delivered to Germany in March 2016 and was distributed to other countries from there: Austria, Belgium, Estonia, France, Luxembourg, Portugal and Switzerland. The spread was available to consumers in special health food stores and via online shops since March 2016.

It has not been possible to demonstrate a link between human cases occurring in Czech Republic, Greece, Serbia and the UK and the distribution of the sesame spread. Austria, Belgium, Estonia, Portugal and Switzerland did not report human cases with this novel serotype even though the same lot of sesame spread that tested positive was distributed there. The sesame spread had also been imported into France and there, the novel serotype was detected in two patients following the defined outbreak period and after the product recall.

The sesame seeds that tested positive in Germany in December 2016 originated from Nigeria where they had been harvested in January 2016. In September 2016, the seeds arrived at company A in Greece, the same company where the sesame spread had been produced 6 months earlier. After processing, the seeds were shipped to a company in Germany via seven deliveries, all of which were part of the same lot. One of these deliveries tested positive for the novel serotype during a company’s own routine check. Investigations by German food safety authorities revealed that all seeds from this delivery were directly sent back to Greece where they were safeguarded by the Greek authorities. Thus, the seeds of that particular delivery never reached the consumer and could not explain any of the cases. The remaining six deliveries originating from the same lot harvested in Nigeria tested negative for *Salmonella* spp. and were further distributed. For these, trace-forward investigations were not undertaken.

**Figure 2:**
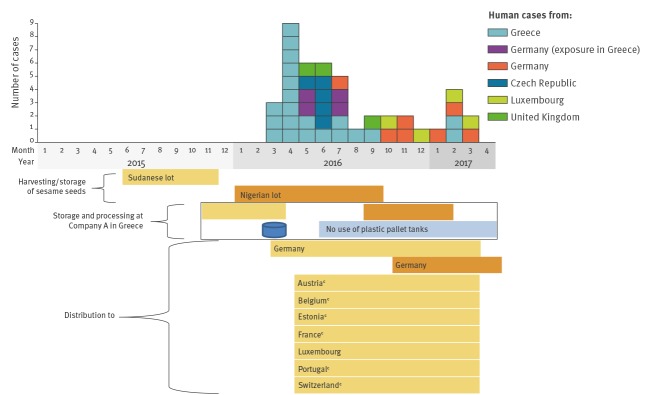
Results of trace-back investigations of sesame spread and Nigerian sesame seeds testing positive for the novel *Salmonella enterica* subspecies *enterica* serotype (11:z41:e,n,z15)^a^ in relation to outbreak cases with known disease onset or date of isolate receipt at the reference laboratory, 1 March 2016–30 April 2017 (n = 44^b^)

^a^ Three food items tested positive for the novel serotype, but only the two with trace-back investigations are reflected in the figure: the sesame spread produced of sesame seeds originating from Sudan and the sesame seeds from Nigeria. There was no trace-back investigation for the third item, the sushi in the United Kingdom for reasons explained in the manuscript.

^b^ Of the 47 outbreak cases, three did not have information regarding disease onset or date of receipt of the isolate at the reference laboratory.

^c^ No known cases during the time of the outbreak.

The results of the tracing investigations are visualised in Figure 2.

Trace-back investigations of the sushi that tested positive for *Salmonella enterica* subspecies *enterica* serotype (antigenic formula 11:z41:e,n,z15) in March 2016 were not carried out in the UK as *Salmonella* spp. was not isolated from any of the 24 constituent ingredients, including three types of sesame seeds. During subsequent attempts to carry out trace-back investigations in 2017, the source of the sesame seeds could not be established.

### Investigations at company A in Greece

The only identified link between the contaminated sesame seeds from Nigeria that were tested in Germany and the sesame spread produced from Sudanese sesame seeds was company A in Greece, a large sesame seed-processing company that distributes seeds and products to various European countries. The Greek authority for food safety visited this company in order to assess the manufacturing processes and hygiene conditions, and to identify possible critical points for cross-contamination. All company A’s checks for *Salmonella* spp. remained negative.

Sesame seeds delivered to company A in Greece undergo the following processing steps before they are directly sold or used as an ingredient in sesame products (RASFF 2017.0221-fup06 [13]): (i) removal of impurities, e.g. stones, dust and plant material, through sieving or other techniques; (ii) washing to remove soil and dust residues; (iii) mechanical hulling to remove the outer skin of the sesame seeds (only relevant for hulled type of sesame seeds); (iv) drying at a minimum of 90 °C, typically 100–105 °C, for 10 min; and (v) sterilising in a sterilisation tower where culinary steam is applied inside a closed spiral tube.

The production line in company A is usually closed. However, during production of the lot of sesame spread that tested positive, the production line was interrupted by an intermediate storage step of the tahini used in the spread. Before the tahini was mixed with the other ingredients of the spread, it was held for 3 days in plastic pallet tanks without documentation of adequate sanitation and appropriate storage conditions. Since July 2016, the mixers have been fed with tahini directly from the pasteuriser through a closed pipeline system, or if there is need for intermediate storage, stainless steel tanks sanitized by a validated sanitation procedure are used (RASFF 2017.0408-fup16 [14]). The Nigerian sesame seeds were processed in September 2016 in company A when the plastic pallet tanks were no longer in use.

## Outbreak control measures

Through interviews of patients in Germany and Luxembourg, a special brand of sesame spread was identified as the probable vehicle of infection, at least in these two countries. The results of the interviews lead to testing of the product. After the sesame spread tested positive for *Salmonella* spp*.* on 28 March 2017, it was recalled from the market at the end of March 2017. Consumers were informed of the recall by written notice in shops that sold the product or by press release. Trace-back investigations of the sesame spread, and of sesame seeds from Nigeria that also tested positive for the novel serotype, identified a company in Greece as the only similarity. This led to an inspection of the production line of sesame products at the company.

## Discussion

Many reports describe salmonellosis outbreaks associated with sesame products involving various diverse serotypes [15-18]. Contamination of sesame seeds or products thereof with *Salmonella* spp. seems to occur regularly. With regards to the detection of *Salmonella* spp. in sesame, 569 notifications have been submitted to RASFF as of 19 August 2019. In addition to notification 2017.0408, which pertains to this investigation, 18 notifications of *Salmonella* spp. in sesame paste, tahini or helva have been submitted to the RASFF portal. The frequent detection of *Salmonella* in sesame products recently motivated studies addressing the survival of *Salmonella* in tahini [19-21], and controls during the production process have been recommended [16]. Possible reasons for contamination, e.g. faecal contamination of irrigation water, need further investigation.

We described a salmonellosis outbreak with 47 confirmed cases across five European countries between March 2016 and April 2017 involving a novel *Salmonella* serotype. The outbreak investigation identified sesame seed products as the vehicle of infection, leading to the recall of a sesame spread and an EU-wide food-chain analysis that suggested cross-contamination between different sesame seed lots in a large sesame seed processing company.

There are several factors that contributed to the success of this outbreak investigation. One, the timely EPIS-FWD notification by Greece underlines the value of international alerts. The outbreak comprised only 47 cases and the temporal and geographical spread of cases outside of Greece resembled that of sporadic cases, which would have made it difficult to detect the outbreak.

Two, the detection of this outbreak was facilitated by the fact that the causative agent was a novel serotype. The same outbreak pattern with a common *Salmonella* serotype, e.g. *S*. Enteritidis, would probably have been detected much later or not at all without high-discrimination typing data. Although the temporally-related occurrence of *Salmonella* isolates of the novel serotype together with the results of the epidemiological studies strongly indicated a direct link between the cases from different countries, the close genetic relatedness of the strains was further confirmed by WGS. This provided important evidence for the comparison of the human strains from different countries and the food isolates.

Three, the rapid sharing of epidemiological information across public health and food safety authorities allowed for timely responses. The results of the case–case study conducted in Greece were communicated early and enabled focused interviews of cases in Germany and Luxembourg. These interviews were pivotal in identifying the sesame spread as one possible outbreak vehicle. This information was directly forwarded to food safety authorities who collected samples of the spread, of which were all from the same lot, the only one available at the time, and tested positive for the novel serotype. The product was consequently recalled from the market.

Four, the positive testing of the food specimens played an important role in supporting the epidemiological evidence. However, taking samples in cases´ households is often not possible or can be realised only with a certain delay. In this case, the long-shelf-life of the product enabled the sampling in cases’ households. For other products, such as eggs, this is often not possible. The analysis of unofficial samples, those purchased from retail in this investigation and not taken directly by food safety authorities, also provided valuable information. Based on this, countries should be encouraged to also share the results of unofficial samples through the appropriate official channel, i.e. RASFF.

Five, it was a lucky coincidence that the *Salmonella* isolate detected in the Nigerian sesame seeds during a company’s own routine check in Germany was serotyped. This is often not the case for food isolates, making it difficult to compare findings from human and food investigations.

Six, the trace-back investigations of the sesame spread made of Sudanese sesame seeds and trace-back investigations of the Nigerian sesame seeds provided strong hints that cross-contamination at company A in Greece was possibly behind this prolonged and European-wide salmonellosis outbreak. A detailed analysis of the production line at company A in Greece in the future could lead to better understanding in assessing critical control points and appropriate measures during the sesame seed processing steps.

Seven, as a consequence of this outbreak investigation, the European Commission included sesame seeds from Nigeria, Sudan and Uganda, the latter because of another RASFF alert regarding *Salmonella* in sesame seeds during that time, to the list of feed and food of non-animal origin subject to an increased level of official controls on imports because of possible *Salmonella* contamination (EU Regulation 2017/1142, effective from 1 July 2017 [22]).

Some questions remain. One, it remains unclear how cross-contamination between the sesame spread and the Nigerian sesame seeds was possible at company A and why sterilisation of the seeds was not effective in preventing salmonellosis infections. Processing of the Sudanese seeds at company A in Greece took place in March 2016. The Nigerian seeds did not arrive at the same company until 6 months later, in September 2016. All sesame seeds are normally washed and heated to more than 90 °C. The only identified irregularity in company A was the use of plastic pallet tanks in March 2016 for which adequate sanitation was not documented. However, these plastic pallet tanks were only used up to June 2016. Therefore, these plastic pallet tanks most likely did not play a role in the outbreak. Neither identifying the reason for the cross-contamination nor the source of contamination (e.g. contaminated irrigation water during the sesame seed production) makes recommending how to avoid both in the future difficult. Whether the initial contamination occurred in Sudan or in another country is impossible to reconstruct on the basis of the available information. There is no information suggesting that the Sudanese seeds were the source of the outbreak. It is possible that the Sudanese seeds were contaminated within company A. No information was available on the origin of the sesame seeds used in the sushi product, which may have been useful in terms of identifying the source of contamination.

The detection of the novel serotype in sushi in the UK was communicated in response to the communication via the statutory EWRS notification system, which was 11 months after the urgent inquiry in EPIS had been launched. At the time, Public Health England (PHE) used the nomenclature ‘*Salmonella* unnamed’ for novel serotypes not yet listed under the White-Kauffmann-Le Minor scheme. This caused a delay in the discovery of the link between the sushi isolate and the outbreak.

After conclusion of the outbreak investigation and the recall of the sesame spread, seven more infections with the novel serotype were detected between May and September 2017. Of these, four were in countries where the sesame spread had been distributed and then recalled (France: n = 2; Germany: n = 1; Luxembourg: n = 1). Possible explanations are that the patients bought the product before the recall at the end of March 2017 and that this long-shelf-life food was still available in their households.

Also, the recall only covered the sesame spread which is likely to not have been the only contaminated product. If cross-contamination between different sesame seed lots was possible at company A, other products produced there could theoretically have been further vehicles of infection. This might also explain the cases and infections in countries where the spread was not distributed: Greece (22 cases, 2 infections in the aftermath); Czech Republic (5 cases); UK (3 cases); and Serbia (1 infection in the aftermath). It might also explain the finding in sushi with light toasted sesame seeds and black sesame seeds. Unfortunately, we do not know which other products may have played a role as vehicles of infection during this outbreak beside the implicated sesame spread and possibly tahini in Greece.

## Lessons learned

There are several factors which could be improved in order to prevent sesame-borne outbreaks in the future. Therefore we would suggest: (i) to carry out further investigations regarding the contamination of sesame seeds and products thereof in order to identify primary contamination sources; (ii) to assess possible places of and conditions for cross-contamination; (iii) to initiate trace-forward investigations on all products made from a contaminated batch of sesame seeds; and (iv) to share all types of information (i.e. from official and unofficial sampling as well as typing results) available on the suspected food through the appropriate channel/system.

This outbreak underlines the importance of an international and cross-sectoral outbreak investigation to identify the causative food vehicle enabling its recall as well as an EU-wide food-chain analysis with the aim to disentangle the origin of the contamination and to raise the awareness of the European Commission resulting in the implementation of risk management measures.

## Supplementary Data

367000 Supplementary Material
