# Supplementary material for: Salmonellosis outbreak with novel Salmonella enterica subspecies enterica serotype (11:z41:e,n,z15) attributable to sesame products in five European countries, 2016 to 2017
Source: Euro Surveill. 2019 Sep 5;24(36):1800543. doi: 10.2807/1560-7917.ES.2019.24.36.1800543 (PMC6737830; doi:10.2807/1560-7917.ES.2019.24.36.1800543)
Supplement: Supplementary Material [file 1800543_SCHIELKE_SupplementaryMaterial.pdf]

Supplementary material

This supplementary material is hosted by Eurosurveillance as supporting information alongside the article [Salmonellosis outbreak with novel Salmonella enterica subspecies enterica serotype (11:z41:e,n,z15) attributable to sesame products in five European countries, 2016 to 2017] on behalf of the authors who remain responsible for the accuracy and appropriateness of the content. The same standards for ethics, copyright, attributions and permissions as for the article apply. Supplements are not edited by Eurosurveillance and Eurosurveillance is not responsible for the maintenance of any links or email addresses provided therein.

Supplementary Table S1: Quality parameters of assembled sequence data used for cgMLST (Ridom SeqSphere+)

Sample ID	Alias ID	ENA-/ NCBI SRA-Accession-No.	ST (*S. enterica* MLST)	Avg. Coverage (Assembled)	Perc. Good Targets (*S. enterica* cgMLST)	#Missing values in Distance Columns
CzR1_human	309	n. a.	2914	102	99.9	3
CzR2_human	366	n. a.	2914	102	99.9	3
CzR3_human	380	n. a.	2914	116	99.9	3
Ger10_human	RKI17-00666	ERS2589580	2914	52	99.9	3
Ger11_human	RKI17-00942	ERS2589579	2914	65	99.9	3
Ger12_human	RKI17-01561	ERS2589577	2914	59	99.9	3
Ger1_human	RKI16-01741	ERS2589588	2914	57	99.7	6
Ger2_human	RKI16-02446	ERS2589587	2914	49	99.6	8
Ger3_human	RKI16-02466	ERS2589586	2914	48	99.7	6
Ger4_human	RKI16-04375	ERS2589585	2914	69	99.8	4
Ger5_human	RKI17-00007	ERS2589583	2914	88	99.7	6
Ger6_human	RKI17-00008	ERS2589582	2914	34	98.7	28
Ger7_human	RKI17-01071	ERS2589578	2914	61	99.9	2
Ger8_human	RKI17-00006	ERS2589584	2914	30	98.2	39
Ger9_human	RKI17-00296	ERS2589581	2914	37	99.2	17
Ger_sesame_seeds	BfR17-SA00239	ERS2589589	2914	50	99.8	5
Ger_sesame_spread	BfR17-SA00773	ERS2589590	2914	41	98.9	24
Greece1_human	81-2016	n. a.	2914	88	99.9	3
Greece2_human	167-2016	n. a.	2914	99	99.9	3
Greece3_human	239-2016	n. a.	2914	107	99.9	3
Lux1_human	17009218	n. a.	2914	89*	99.9	3
Lux2_human	16055570	n. a.	2914	27*	97.2	60
Lux3_human	16063866	n. a.	2914	50*	99.2	18
Lux_sesame_spread	17018901	n. a.	2914	96*	99.9	3
UK1_human	265329	SRR5413121	2914	73	99.7	6
UK2_human	253443	SRR5413119	2914	83	99.7	6
UK3_human	301640	SRR5413117	2914	60	99.5	11
UK_sushi	235122	SRR5413122	2914	39	96.5	76

n. a. = not available *from Enterobase Assembly Stats
